# Prognostic value of aneurysmal subarachnoid hemorrhage scores on mortality and disability in a Brazilian tertiary center

**DOI:** 10.1055/s-0046-1818608

**Published:** 2026-05-12

**Authors:** Thire Baggio Machado Marazzi, Thiago Oscar Goulart, Brunna Pileggi Rimoli, Octávio Marques Pontes-Neto

**Affiliations:** 1 Universidade de São Paulo, Faculdade de Medicina de Ribeirão Preto, Departamento de Neurociências e Ciências do Comportamento, Ribeirão Preto SP, Brazil.; 2 Harvard University, Harvard T.H. Chan School of Public Health, Department of Epidemiology, Boston MA, United States.; 3 University of Toronto, Temerty Faculty of Medicine, Department of Neurology, Toronto ON, Canada.

**Keywords:** Subarachnoid Hemorrhage, Prognosis, Hospital Mortality

## Abstract

**Background:**

Aneurysmal subarachnoid hemorrhage (aSAH) is a severe neurological emergency associated with high rates of mortality and disability. While multiple prognostic scores have been developed in high-income countries, evidence from low- and middle-income settings remains limited.

**Objective:**

To evaluate the predictive performance of clinical and radiological scores for mortality and functional outcomes in aSAH patients treated in a public tertiary center in a resource-limited country.

**Methods:**

We conducted a retrospective cohort study of adult patients with confirmed aSAH admitted between June 2018 and March 2022. Eight prognostic scores were applied: World Federation of Neurosurgical Societies (WFNS), Barrow Neurological Institute (BNI), VASOGRADE, Hunt and Hess score, Age, Intraventricular hemorrhage, and Rebleeding (HAIR), WFNS grade, Age, and Pupillary reactivity (WAP), Hemorrhage, Age, Treatment, Clinical Status, and Hydrocephalus (HATCH), Brain Aneurysm Institute (BAI), and modified BNI score. Primary outcomes were in-hospital mortality and poor functional outcome (modified Rankin Scale 4–6) at 90 days. Discriminative ability was assessed using the area under the receiver operating characteristic curve (AUROC) and bootstrap comparisons.

**Results:**

Among 74 patients, in-hospital mortality was 42%, and 68.9% had poor functional outcomes. All scores demonstrated good predictive performance (AUROC ≥ 0.78). The mBNI and WFNS had the highest AUROCs for functional outcome (0.88 and 0.86, respectively), with mBNI significantly outperforming BNI (difference = 0.16; 95% CI: 0.085–0.25). The HATCH score showed moderate accuracy (AUROC 0.79), although significantly inferior to mBNI in pairwise comparison. There were no missing data, and scores were not used to guide clinical care.

**Conclusion:**

Despite being developed in high-income countries, the selected prognostic scores showed strong performance in a resource-limited setting. These results support their use as early risk stratification tools and emphasize the need for further validation in middle-income healthcare systems.

## INTRODUCTION

Aneurysmal subarachnoid hemorrhage (aSAH) remains a devastating neurological emergency, often affecting relatively young individuals and leading to high rates of mortality and long-term disability. 1 2 3 4 While advances in early diagnosis, surgical or endovascular treatment, and intensive care have substantially improved outcomes in high-income countries, similar progress has not been uniformly observed in low- and middle-income countries (LMICs), where limited resources and access barriers can hinder timely and comprehensive care.

Accurate early prognostication is essential for clinical decision-making, appropriate triage, communication with families, and optimizing the use of healthcare resources. Over the past decades, numerous clinical and radiological scoring systems have been developed to predict outcomes in aSAH, including the World Federation of Neurosurgical Societies (WFNS) scale, 5 modified Fisher scale, 6 Barrow Neurological Institute (BNI) score, 7 VASOGRADE, 8 Hunt and Hess score, Age, Intraventricular hemorrhage, and Rebleeding (HAIR), 9 WFNS grade, Age, and Pupillary reactivity (WAP), 10 Hemorrhage, Age, Treatment, Clinical Status, and Hydrocephalus (HATCH), 11 and Brain Aneurysm Institute (BAI). 12 These tools aim to estimate the likelihood of death or functional disability, and several have demonstrated good discriminatory performance in different settings.

However, most of these scores were created and validated in high-income countries, often under better conditions that may not reflect the complexities and constraints of LMIC healthcare environments. As such, their external validity and real-world applicability in resource-limited contexts remain uncertain.

The present study aims to assess the predictive performance of eight commonly used prognostic scores in a cohort of patients with aSAH treated at a tertiary public hospital in Brazil. By evaluating their accuracy for predicting in-hospital mortality and functional outcomes at 90 days, we seek to explore their utility in middle-income healthcare settings and contribute to global efforts to validate and adapt prognostic tools across diverse populations.

### Clinical and radiological prognostic scales

Historically, the modified Fisher scale (mFisher) 6 is the most widely used radiological tool for outcome prediction after aSAH, while the Hunt and Hess (HH) scale 13 remains the most traditional clinical grading system. Despite their widespread use, both scores rely on somewhat subjective interpretations, which has led to significant inter-rater variability. In response, more objective and quantifiable scoring systems have been proposed and validated in recent years.

The BNI grading scale is a radiological score originally developed to predict delayed cerebral ischemia (DCI) with greater accuracy than the mFisher scale. 7 Neidert et al. later validated the BNI scale in a multicenter cohort, demonstrating a significant association between higher BNI grades and poor functional outcomes. 14 However, the original BNI scale demonstrated limited predictive accuracy for favorable outcomes, with an AUC of 0.66. To enhance its prognostic performance, the authors proposed a modified BNI score, combining the BNI grade with the WFNS 5 scale and age over 60 years, which increased the AUC to 0.768 ( *p* < 0.001). For clarity throughout this manuscript, this version will be referred to as the “modified BNI” (mBNI) score. Both the original and mBNI scores were included in this study for comparative evaluation within the Brazilian population.

As for clinical scoring systems, the WFNS and WAP scales were selected. The WFNS score, introduced in 1988, integrates the Glasgow Coma Scale (GCS) and focal neurological deficits to stratify patients in a straightforward and reproducible manner. 5 In 2018, Zheng et al. identified 3 key predictors of functional outcome (WFNS score, age, and pupillary dilation) and proposed the WAP scale as a mnemonic tool based on these factors. 10 Both clinical scales were incorporated into the present study.

Finally, we included four composite scores that integrate both clinical and radiological variables: VASOGRADE, 8 BAI, 12 HAIR, 9 and HATCH. 11 The VASOGRADE scale was developed to estimate the risk of DCI by integrating WFNS and mFisher data. The BAI score, proposed in 2018, aims to predict mortality and functional outcomes at discharge using readily accessible clinical information. 12 A subsequent study led to the creation of the HATCH score, which incorporates additional variables collected within the first 24 hours of hospital admission to improve prediction of 1-year outcomes and decompression illness (DCI) risk. 11 The HAIR score, 9 proposed in 2014, uses a mnemonic (Hunt Hess score, 13 Age, Intraventricular hemorrhage, and Rebleeding) to predict in-hospital mortality, and was later validated by Abulhasan et al. in 2018. 15

All selected scores had demonstrated at least moderate correlation with their respective outcomes in the original validation studies. Table 1 summarizes the components and characteristics of each scale included in our analysis.

**Table 1:** Variables analyzed per selected score

Score	Variables	Reference
BAI	Age, antiaggregant drug use, Glasgow coma scale, maximum thickness subarachnoid hemorrhage (mm)	12
BNI	Maximum thickness subarachnoid hemorrhage (mm)	7
Modified BNI	Age, BNI score, WFNS	14
HAIR	Hunt Hess scale, age, intraventricular hemorrhage, rebleed	9
HATCH	Maximum thickness subarachnoid hemorrhage (mm), age, treatment (clipping), occurrence of hydrocephalus, rebleeding	11
VASOGRADE	Modified Fisher scale, WFNS	8
WAP	WFNS, Age, pupilar exam	10
WFNS	Focal deficit, Glasgow coma scale	5

Abbreviations: BAI, Brain Aneurysm Institute Scale; BNI, Barrow Neurological Institution Grade Scaling; HAIR, Hunt Hess scale, age, intraventricular hemorrhage, rebleed; HATCH, hemorrhage, age, treatment, clinical state, hydrocephalus score; WAP, WFNS grade, Age, and Pupillary reactivity; WFNS, World Federation of Neurosurgical Societies.

## METHODS

The current retrospective, single-center observational study included adult patients with a confirmed diagnosis of aSAH who were consecutively admitted to the Emergency Department of Hospital de Clínicas da Faculdade de Medicina da Universidade de São Paulo em Ribeirao Preto (HCFMUSP-RP) between June 2018 and March 2022. The study period was defined based on 2 institutional milestones: the digitization of the Registro de Acidente Vascular Encefálico de Ribeirão Preto (REAVER) platform in 2018 and the initiation of standardized institutional care protocols for aSAH in 2022.

Since 2014, the REAVER registry has systematically recorded data on patients admitted with cerebrovascular diseases, including demographic, clinical, radiological, and functional outcomes. Data is collected prospectively and independently of the attending team (Neurology, Neurosurgery, or Interventional Neuroradiology), ensuring a comprehensive institutional dataset.

Patients were eligible for inclusion if they were 18 years or older, had a diagnosis of SAH confirmed by either cranial computed tomography (CT) or lumbar puncture, and had a documented aneurysmal etiology confirmed by CT angiography or digital subtraction angiography. To ensure sufficient clinical documentation for outcome analysis, only patients admitted within five days of ictus and those who remained hospitalized at HCFMUSP-RP were included. Patients transferred solely for aneurysm treatment were excluded.

Demographic and clinical data, including sex, age, comorbidities, chronic medication use, and clinical presentation, were extracted retrospectively from the institutional electronic medical record system and stored in a secure research database with restricted access to the principal investigator.

Radiological evaluation included classification according to the modified Fisher scale, assessment of intraventricular hemorrhage (IVH), and measurement of maximum subarachnoid hemorrhage thickness (in millimeters), following the standardized protocol described by Wilson et al. 7

The 3-month modified Rankin Scale (mRS) score was obtained through structured telephone interviews conducted by trained members of the REAVER registry team.

### Scoring system selection

Several prognostic scales for aneurysmal subarachnoid hemorrhage (aSAH) have been previously developed and validated. Scales were selected as a priori based on predefined criteria: Established use in aSAH literature; applicability to initial clinical and radiological data; and inclusion in prior comparative or validation studies.

Only scales that assessed in-hospital mortality or functional outcome were considered. We excluded tools that required laboratory biomarkers, advanced imaging postprocessing, or proprietary software not routinely available in standard clinical settings.

### Statistical analysis

All statistical analyses were performed using the IBM SPSS Statistics for Windows software (IBM Corp.), version 21.0, and Python (Scikit-learn and NumPy libraries). Descriptive statistics were used to characterize demographic, clinical, and radiological variables.

The prognostic performance of each score was assessed through receiver operating characteristic (ROC) curve analysis, with calculation of the area under the receiver operating characteristic curve (AUROC) for 2 primary outcomes: in-hospital mortality and functional disability at 3-month follow-up (defined as modified Rankin Scale score of 4–6). Values of AUROC closer to 1.0 were interpreted as indicating stronger discriminatory ability, while values near 0.5 indicated no predictive power. Pairwise comparisons between AUROCs were performed using nonparametric bootstrapping with 2,000 replicates. Statistical significance was defined as *p* < 0.05.

There were no missing data for score components or outcomes. Prognostic scores were calculated retrospectively and did not influence clinical decisions or care limitations. Although intensive care unit (ICU) resources are limited at our institution, there were no cases of treatment withdrawal or withholding of care based on the prognostic scores.

The present work was supported by generative artificial intelligence tools only to assist with scientific writing and language revision. All decisions regarding content, data analysis, and interpretation were made by the authors, with human oversight at every stage.

### Ethical considerations

Ethical approval for the study was obtained from the local ethics committee (registration CAAE: 63926022.9.0000.5440). Informed consent was initially obtained for participation in the clinical data registry platform from which all patient data were retrospectively extracted. The dataset was anonymized prior to analysis, and all procedures were conducted in accordance with the Declaration of Helsinki and national regulatory guidelines.

## RESULTS

### Patient selection

**Figure 1:**
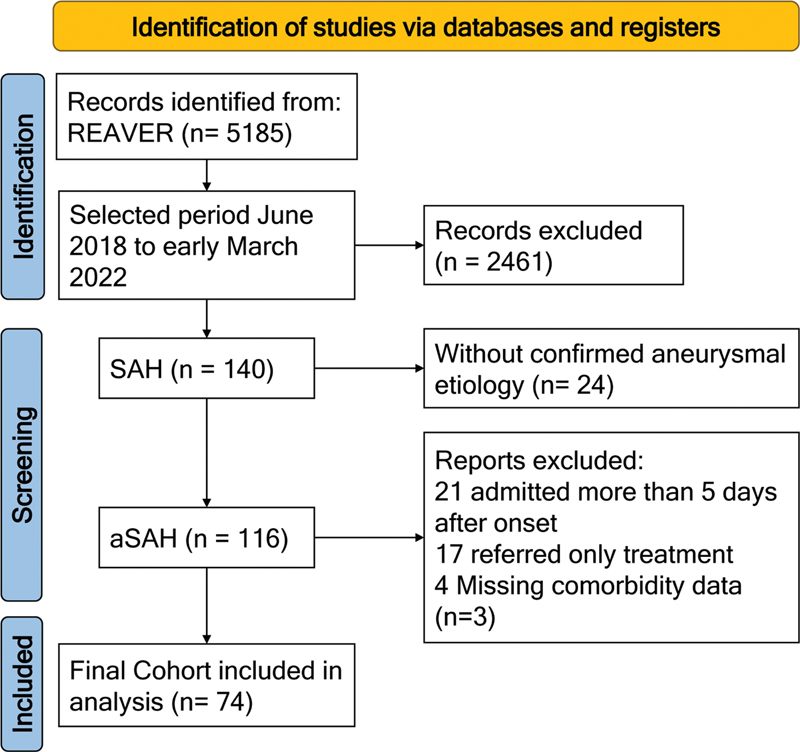
Notes: REAVER platform: Registro de Acidente Vascular Encefálico de Ribeirão Preto, a local registry of patients with cerebrovascular diseases maintained by academic hospitals in Ribeirão Preto, SP, Brazil. Flow diagram for the selection of patients with aneurysmal subarachnoid hemorrhage (aSAH) from the REAVER database.

Patient selection is summarized in Figure 1 . A total of 5,185 cases were identified in the REAVER registry between June 2018 and March 2022. Of these, 140 patients were initially classified as SAH. After excluding cases without confirmed aneurysmal etiology (n = 24), and additional exclusions based on late admission (n = 21), referral for treatment only (n = 17), or incomplete comorbidity data (n = 3), a final cohort of 74 patients with aSAH was included.

### Patient characteristics

A total of 74 patients with confirmed aSAH were included in the final analysis. The mean age was 56.6 ± 14.3 years, and 72% were women. Most patients (93%) had no prior disability (mRS: 0) before the hemorrhagic event. On admission, focal neurological deficits were observed in 34% of cases, hydrocephalus in 64%, pupillary dilation in 16%, and rebleeding in 12%. Most patients presented with high-grade WFNS scores: 45% were classified as WFNS 4 or 5.

Radiologically, 73% of patients were classified as modified Fisher 4, and 42% had intraventricular hemorrhage (IVH). The mean maximum SAH thickness was 8 ± 7.1 mm. Regarding treatment, 19% underwent surgical clipping. The in-hospital mortality rate was 41%, and 69% had poor functional outcomes (mRS: 4–6) at 3 months. Demographic, clinical, and radiological characteristics are described in Table 2 .

**Table 2:** Demographic, clinical, and radiological characteristics of patients with aneurysmal subarachnoid hemorrhage (n = 74)

Variable	Value
Age (years)	Mean: 56.6 ± 14.3; range: 21–87
Female gender: n (%)	54 (72%)
Modified Rankin Scale before SAH: n (%)	mRS 0: 69 (93%) mRS 1–3: 5 (7%)
Clinical presentation: n (%)	
Focal neurological deficits: n (%)	25 (34%)
Acute hydrocephalus: n (%)	47 (64%)
Pupillary dilatation: n (%)	12 (16%)
Rebleeding prior to admission: n (%)	9 (12%)
WFNS score on admission: n (%)	I: 15 (20%) II: 19 (26%) III: 7 (9%) IV: 11 (15%) V: 22 (30%)
Use of antiplatelet agents: n (%)	10 (14%)
Radiological findings	
Modified Fisher scale: n (%)	0: 1 (1%) 1: 3 (4%) 2: 4 (5%) 3: 12 (16%) 4: 54 (73%)
Maximum subarachnoid blood thickness (mm)	Mean: 8.0 ± 7.1; range: 0–32
Intraventricular hemorrhage: n (%)	31 (42%)
Treatment: n (%)	
Aneurysm clipping: n (%)	14 (19%)
In-hospital mortality: n (%)	31 (42%)
Modified Rankin Scale at 3 months: n (%)	0–2 (independent): 22 (30%) 3: 3 (4%) 4–5: 18 (24%) 6 (death): 31 (42%)

Abbreviations: SHA, subarachnoid hemorrhage; WFNS, World Federation of Neurosurgical Societies.

### Predictive performance of clinical scores

All 8 prognostic scores demonstrated good to excellent discrimination for both in-hospital mortality and poor functional outcome (mRS > 3 at 3 months), with AUROC values above 0.78. The mBNI score showed the highest predictive accuracy for both outcomes (AUROC: 0.88 for functional outcome; 95%CI: 0.81–0.95; and AUROC: 0.86 for in-hospital mortality; 95%CI: 0.78–0.93), followed by the WFNS (AUROC: 0.86 for functional outcome; 95%CI: 0.78–0.93). The HATCH score also demonstrated consistent performance, with AUROC 0.78 for in-hospital mortality and 0.79 for poor functional outcome, indicating moderate discriminative ability in this cohort. Table 3 summarizes the discriminative performance of each prognostic score for predicting both in-hospital mortality and poor functional outcome at 90 days (mRS: 4–6).

**Table 3:** Prognostic performance of clinical scores for in-hospital mortality and functional outcome at 3 months

Score	In-hospital mortality: AUROC (95%CI)	Functional outcome at 3 months: AUROC (95%CI)
mBNI	0.86 (0.78–0.93)	0.88 (0.81–0.95)
WFNS	0.85 (0.76–0.92)	0.86 (0.78–0.93)
VASOGRADE	0.83 (0.73–0.91)	0.84 (0.76–0.92)
HAIR	0.81 (0.70–0.89)	0.83 (0.75–0.91)
WAP	0.80 (0.70–0.88)	0.81 (0.72–0.89)
BAI	0.79 (0.69–0.87)	0.80 (0.70–0.88)
HATCH	0.78 (0.67–0.87)	0.79 (0.69–0.87)
BNI	0.76 (0.65–0.85)	0.72 (0.60–0.82)

Abbreviations: AUC, area under curve; AUROC, area under the receiver operating characteristic curve; BAI, Brain Aneurysm Institute Scale; BNI, Barrow Neurological Institution Grade Scaling; CI, confidence interval; HAIR, Hunt Hess scale, age, intraventricular hemorrhage, rebleed; HATCH, hemorrhage, age, treatment, clinical state, hydrocephalus score; WAP, WFNS grade, Age, and Pupillary reactivity; WFNS, World Federation of Neurosurgical Societies.

When comparing AUROCs for functional outcome ( Table 4 ), mBNI performed significantly better than BNI (difference = 0.16; 95%CI: 0.085–0.25; *p* < 0.001). Other scores had overlapping confidence intervals, suggesting similar predictive performance. For example, VASOGRADE (AUROC: 0.84; 95%CI: 0.76–0.92) and HAIR (AUROC: 0.83; 95%CI: 0.75–0.91) performed comparably. In pairwise comparison, the mBNI also outperformed the HATCH score for functional outcome prediction (difference = 0.09; 95%CI: 0.01–0.17; *p* = 0.04), while HATCH and WFNS showed no significant difference (difference = 0.03; 95%CI: −0.04–0.09).

**Table 4:** Pairwise comparison of AUROC values between prognostic scores

Score 1	Score 2	Difference in AUROC	95%CI	*p* -value
mBNI	BNI	0.16	0.085–0.25	< 0.001
mBNI	HATCH	0.09	0.01–0.17	0.04
WFNS	BNI	0.1	0.02–0.18	0.01
WFNS	HATCH	0.03	−0.04–0.09	0.30
WFNS	VASOGRADE	0.05	−0.03–0.13	0.22
WFNS	HAIR	0.04	−0.03–0.11	0.25

Abbreviations: AUC, area under curve; AUROC, area under the receiver operating characteristic curve; BAI, Brain Aneurysm Institute Scale; BNI, Barrow Neurological Institution Grade Scaling; HAIR, Hunt Hess scale, age, intraventricular hemorrhage, rebleed; HATCH, hemorrhage, age, treatment, clinical state, hydrocephalus score; WAP, WFNS grade, Age, and Pupillary reactivity; WFNS, World Federation of Neurosurgical Societies.

**Figure 2:**
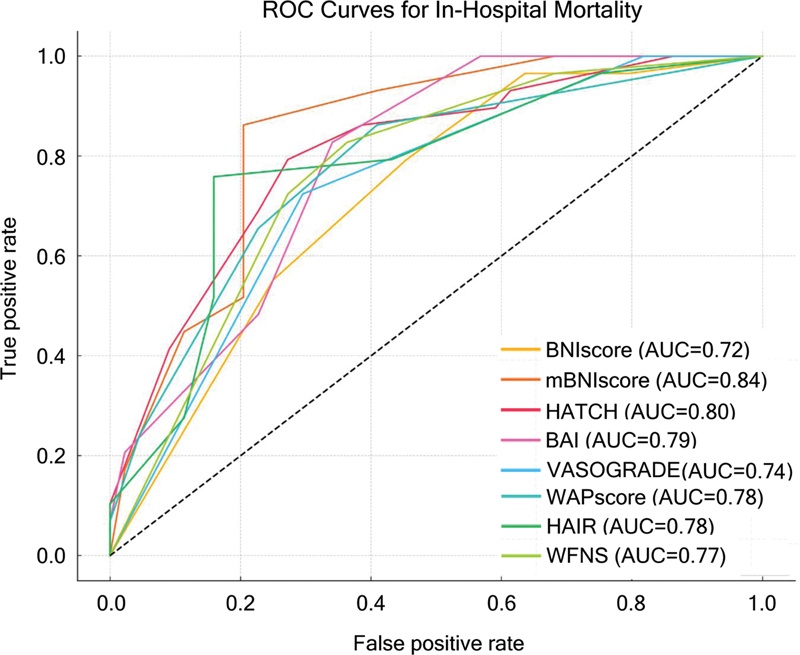
Abbreviations: AUC, area under curve; BNI, Barrow Neurological Institution Grade Scaling; WFNS, World Federation of Neurosurgical Societies; BAI, Brain Aneurysm Institute Scale; HAIR, Hunt Hess scale, age, intraventricular hemorrhage, rebleed; HATCH, hemorrhage, age, treatment, clinical state, hydrocephalus score; WAP, WFNS grade, Age, and Pupillary reactivity. Predictive scores and in-hospital mortality receiver operating characteristic curves and C-statistics.

For in-hospital mortality, although mBNI also ranked highest, no statistically significant differences were observed in pairwise comparisons, likely due to the overlapping 95%CIs. None of the scores presented AUROC values close to 0.5, reinforcing that all tested tools retain at least moderate prognostic utility in this population. To illustrate these findings, ROC curves for all scores are shown in Figures 2 3 .

**Figure 3:**
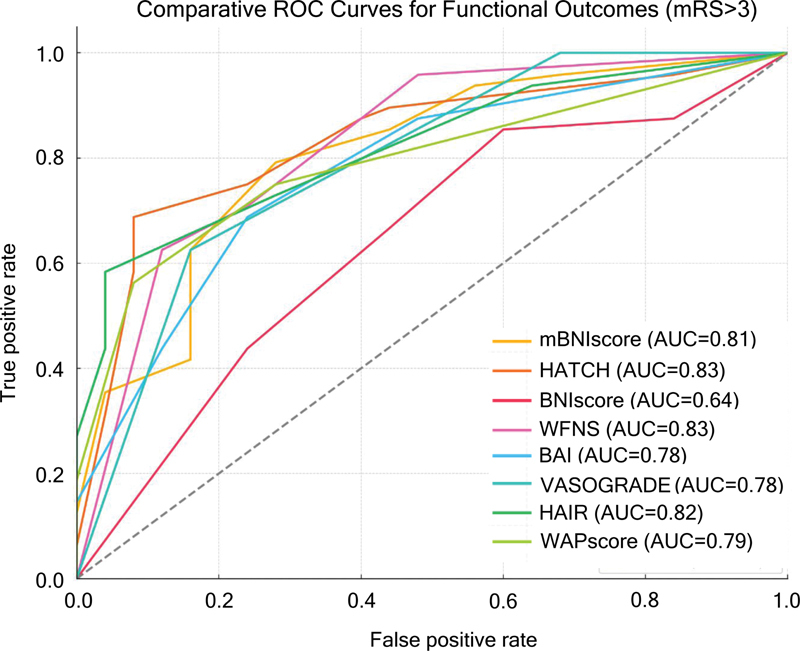
Abbreviations: AUC, area under curve; BNI, Barrow Neurological Institution Grade Scaling; WFNS, World Federation of Neurosurgical Societies; BAI, Brain Aneurysm Institute Scale; HAIR, Hunt Hess scale, age, intraventricular hemorrhage, rebleed; HATCH, hemorrhage, age, treatment, clinical state, hydrocephalus score; WAP, WFNS grade, Age, and Pupillary reactivity. Predictive scores and disability in 3 months receiver operating characteristic curves and C-statistics.

## DISCUSSION

The present study evaluated the prognostic performance of eight clinical and radiological scores in predicting in-hospital mortality and functional outcome at 3 months in a cohort of Brazilian patients with aSAH treated at a public tertiary academic hospital. The institution is maintained by the Brazilian Unified Health System (Sistema Único de Saúde – SUS, in Portuguese), a publicly funded universal healthcare system. Considering that approximately two thirds of the Brazilian population rely exclusively on SUS, 16 the clinical profile and outcomes observed in this cohort are highly representative of real-world conditions in low- and middle-income settings, particularly in public hospitals.

In this context, we observed in-hospital mortality of 42% and functional dependence (mRS 4–6) in 69% of patients at 90 days. These findings indicate a high burden of disease and are comparable to results from other Brazilian hospital-based studies. In the cohort described by Souza et al., 17 18 in-hospital mortality was 39.6% (n = 67/169), while Kurtz et al. 19 reported mortality of 42.3% (n = 85/201) across multiple centers. Such high rates likely reflect a severity bias, as our sample included a substantial proportion of patients with WFNS grade IV or V at presentation (45.9%), a known predictor of poor prognosis.

An important contextual consideration is that prognostic scores at admission were not employed as a basis for decisions regarding treatment limitation in our setting. In our cohort, although some patients may have progressed to unfavorable outcomes, there were no documented cases in which prognostic scales were explicitly used to justify the withdrawal or withholding of life-sustaining therapy. This observation aligns with the findings of the multicenter study by Kurtz et al., 19 which reported that only 9.4% of patients had any documented limitation of therapeutic support. This systemic characteristic must be considered when interpreting outcomes and comparing them to studies conducted in countries where early prognostication may influence decisions regarding treatment escalation or withdrawal.

Despite the clinical importance of early risk stratification, few studies in Brazil have examined the comparative performance of prognostic scores in aSAH. One of the few is the study which evaluated the VASOGRADE score in a Brazilian cohort. 17 Our study expands upon this by directly comparing eight validated prognostic tools within the same population, providing new insights into their relative performance in a middle-income public healthcare setting.

Among the evaluated scores, the BNI 14 and WFNS 5 demonstrated the highest discriminatory ability for predicting functional outcomes at 90 days in our cohort, with AUROCs of 0.88 and 0.86 respectively. In contrast, the original BNI score 7 had a slightly lower AUROC of 0.84, with no statistically significant difference when compared to the mBNI (AUROC difference: 0.04; 95%CI: –0.03–0.12; *p* = 0.28). This differs from the results reported by Neidert et al., 14 who originally proposed the mBNI classification. In their study, conducted in Switzerland and Germany with 312 patients, the mBNI showed a significantly improved predictive performance over the original BNI (AUROC: 0.84 versus 0.71: *p* < 0.001), particularly for unfavorable functional outcomes at discharge and at 6 months. These results supported the idea that subdividing high-volume bleeds provides better discrimination within the higher-risk spectrum. However, our findings suggest that this incremental gain may not be universally reproducible, possibly due to sample size, population differences, or the already high prevalence of poor-grade hemorrhages in our cohort.

The HATCH score also showed moderate discriminative accuracy in this population (AUROC: 0.78–0.79), performing similarly to WFNS in pairwise analysis and remaining clinically informative despite not surpassing the performance of the mBNI. These findings support the utility of HATCH as an accessible composite tool, while highlighting that its incremental value over traditional scores may be limited in cohorts with high baseline severity.

The WFNS score, although developed decades ago, remains one of the most widely used grading systems in aSAH. Despite its age, it demonstrated robust predictive value in our cohort, with performance comparable to that of the mBNI. This reinforces its clinical utility, especially in resource-limited settings where it remains simple to apply and reliably reflects initial neurological status, a major determinant of prognosis. In our study, WFNS was the only scale significantly associated with both mortality and functional disability on univariate analysis.

The current study has limitations. The single-center design and relatively small sample size limit generalizability and preclude multivariate analysis or formal AUROC comparisons. The retrospective nature also introduces potential biases related to data completeness and heterogeneity in care delivery. Nevertheless, the results provide valuable insight into prognostic evaluation in a context representative of the broader SUS network and other public health systems in resource-limited regions.

Another limitation is that the prediction of favorable functional outcomes could not be assessed, as only a small proportion of patients achieved independence at 90 days. Because of this low event rate, any discrimination analysis would have produced unstable estimates. Therefore, we focused on poor functional outcome (mRS: 4–6), which was sufficiently frequent and clinically meaningful in this high-severity cohort.

In summary, the present study contributes novel evidence regarding the utility of established aSAH prognostic scores in the Brazilian public healthcare setting. The strong performance of accessible, low-complexity scores such as mBNI and WFNS suggests that their systematic use may improve early risk assessment and guide clinical decision-making in environments like ours.

In this study, conducted in a Brazilian public hospital integrated into SUS, we observed high rates of in-hospital mortality and functional disability among patients with aSAH. The prognostic scores evaluated, particularly the modified BNI, HATCH, and WFNS, demonstrated strong predictive accuracy for both mortality and disability, reinforcing their utility even in resource-limited environments.

These tools are simple and based on routinely available clinical and imaging data, making them highly suitable for implementation in middle-income healthcare settings such as Brazil. Their systematic use may enhance early risk stratification, clinical decision-making, and outcome monitoring in public neurocritical care services.

## References

[1] Chou S HY Subarachnoid Hemorrhage Continuum (Minneap Minn) 2021 27 05 1201 1245 10.1212/CON.0000000000001052 34618758

[2] Seule M Oswald D Muroi C Brandi G Keller E Outcome, Return to Work and Health-Related Costs After Aneurysmal Subarachnoid Hemorrhage Neurocrit Care 2020 33 01 49 57 10.1007/s12028-019-00905-2 31919809

[3] Le Roux A A Wallace M C Outcome and cost of aneurysmal subarachnoid hemorrhage Neurosurg Clin N Am 2010 21 02 235 246 10.1016/j.nec.2009.10.014 20380966

[4] Fernando S M Reardon P M Dowlatshahi D Outcomes and costs of patients admitted to the ICU due to spontaneous intracranial hemorrhage Crit Care Med 2018 46 05 e395 e403 10.1097/CCM.0000000000003013 29406421

[5] Rosen D S Macdonald R L Subarachnoid hemorrhage grading scales: a systematic review Neurocrit Care 2005 2 02 110 118 10.1385/NCC:2:2:110 16159052

[6] Lindvall P Runnerstam M Birgander R Koskinen L OD The Fisher grading correlated to outcome in patients with subarachnoid haemorrhage Br J Neurosurg 2009 23 02 188 192 10.1080/02688690802710668 19306176

[7] Wilson D A Nakaji P Abla A A A simple and quantitative method to predict symptomatic vasospasm after subarachnoid hemorrhage based on computed tomography: beyond the Fisher scale Neurosurgery 2012 71 04 869 875 10.1227/NEU.0b013e318267360f 22801639

[8] SAHIT collaborators Manoel ALdO Jaja B N Germans M R The VASOGRADE: A Simple Grading Scale for Prediction of Delayed Cerebral Ischemia After Subarachnoid Hemorrhage Stroke 2015 46 07 1826 1831 10.1161/STROKEAHA.115.008728 25977276

[9] Lee V H Ouyang B John S Risk stratification for the in-hospital mortality in subarachnoid hemorrhage: the HAIR score Neurocrit Care 2014 21 01 14 19 10.1007/s12028-013-9952-9 24420695

[10] Zheng K Zhong M Zhao B Poor-grade aneurysmal subarachnoid hemorrhage: Risk factors affecting clinical outcomes in intracranial aneurysm patients in a multi-center study Front Neurol 2019 10(FEB):123 10.3389/fneur.2019.00123 30873104 PMC6400833

[11] Hostettler I C Sebök M Ambler G Validation and Optimization of Barrow Neurological Institute Score in Prediction of Adverse Events and Functional Outcome after Subarachnoid Hemorrhage—Creation of the HATCH (Hemorrhage, Age, Treatment, Clinical State, Hydrocephalus) Score Neurosurgery 2020 88 01 96 105 10.1093/neuros/nyaa316 32779716

[12] Maragkos G A Enriquez-Marulanda A Salem M M Proposal of a Grading System for Predicting Discharge Mortality and Functional Outcome in Patients with Aneurysmal Subarachnoid Hemorrhage World Neurosurg 2019 121 e500 e510 10.1016/j.wneu.2018.09.148 30268551

[13] Hunt W E Hess R M Surgical risk as related to time of intervention in the repair of intracranial aneurysms J Neurosurg 1968 28 01 14 20 10.3171/jns.1968.28.1.0014 5635959

[14] Swiss SOS study group Neidert M C Maldaner N Stienen M N The Barrow Neurological Institute Grading Scale as a Predictor for Delayed Cerebral Ischemia and Outcome After Aneurysmal Subarachnoid Hemorrhage: Data From a Nationwide Patient Registry (Swiss SOS) Neurosurgery 2018 83 06 1286 1293 10.1093/neuros/nyx609 29351673

[15] Abulhasan Y B Alabdulraheem N Simoneau G Angle M R Teitelbaum J Mortality after Spontaneous Subarachnoid Hemorrhage: Causality and Validation of a Prediction Model World Neurosurg 2018 112 e799 e811 10.1016/j.wneu.2018.01.160 29410174

[16] Paim J Travassos C Almeida C Bahia L Macinko J The Brazilian health system: history, advances, and challenges Lancet 2011 377 9779 1778 1797 10.1016/S0140-6736(11)60054-8 21561655

[17] Souza NVdO Rouanet C Solla D JF The Role of VASOGRADE as a Simple Grading Scale to Predict Delayed Cerebral Ischemia and Functional Outcome After Aneurysmal Subarachnoid Hemorrhage Neurocrit Care 2023 38 01 96 104 10.1007/s12028-022-01577-1 36002635

[18] Souza NVdO Rouanet C Solla D JF Impact of Medical and Neurologic Complications on the Outcome of Patients with Aneurysmal Subarachnoid Hemorrhage in a Middle-Income Country World Neurosurg 2024 183 e250 e260 10.1016/j.wneu.2023.12.068 38104933

[19] Kurtz P Taccone F S Bozza F A Systemic Severity and Organ Dysfunction in Subarachnoid Hemorrhage: A Large Retrospective Multicenter Cohort Study Neurocrit Care 2021 35 01 56 61 10.1007/s12028-020-01139-3 33150574

